# Novel insights into post-myocardial infarction cardiac remodeling through algorithmic detection of cell-type composition shifts

**DOI:** 10.1371/journal.pgen.1011807

**Published:** 2025-07-24

**Authors:** Brian Gural, Logan Kirkland, Abigail Hockett, Peyton Sandroni, Jiandong Zhang, Manuel Rosa-Garrido, Samantha K. Swift, Douglas J. Chapski, Michael A. Flinn, Caitlin C. O’Meara, Thomas M. Vondriska, Michaela Patterson, Brian C. Jensen, Christoph D. Rau

**Affiliations:** 1 Department of Genetics and Computational Medicine Program, UNC School of Medicine, University of North Carolina at Chapel Hill, Chapel Hill, North Carolina, United States of America; 2 McAllister Heart Institute, UNC School of Medicine, University of North Carolina at Chapel Hill, Chapel Hill, North Carolina, United States of America; 3 Department of Medicine, Division of Cardiology, UNC School of Medicine, University of North Carolina at Chapel Hill, Chapel Hill, North Carolina, United States of America; 4 Department of Pharmacology, UNC School of Medicine, University of North Carolina at Chapel Hill, Chapel Hill, North Carolina, United States of America; 5 Department of Cellular and Molecular Signaling, The Institute of Biomedicine and Biotechnology of Cantabria, Santander, Spain; 6 Department of Cell Biology, Neurobiology, and Anatomy, Medical College of Wisconsin, Milwaukee, Wisconsin, United States of America; 7 Departments of Anesthesiology & Perioperative Medicine, Medicine/Cardiology, and Physiology, David Geffen School of Medicine; Molecular Biology Institute; University of California, Los Angeles, California, United States of America; 8 Department of Physiology, Medical College of Wisconsin, Milwaukee, Wisconsin, United States of America; University College London, UNITED KINGDOM OF GREAT BRITAIN AND NORTHERN IRELAND

## Abstract

Interpreting bulk RNA sequencing from heterogeneous tissues like the post-myocardial infarction (MI) heart is confounded by dynamic changes in cell-type composition. To address this, we developed a computational approach using single-nucleus RNA sequencing (snRNA-seq) references to estimate and correct for cell-type abundance shifts in bulk transcriptomic data. We applied this method to analyze infarct border zone transcriptomes from wild-type (WT) and cardiomyocyte-specific α1A-adrenergic receptor knockout (cmAKO) mice subjected to MI via left coronary artery ligation or sham surgery. Our analysis revealed exaggerated cardiomyocyte loss and fibroblast gain in cmAKO mice post-MI compared to WT, implicating α1A-ARs in maintaining cellular homeostasis. We then demonstrate the confounding effect of composition changes though simulations: a modest 10% change in the major cell type’s abundance caused over 20% of transcripts to appear as differentially expressed genes (DEGs) when composition was ignored. Applying our correction method refined the interpretation of MI-induced transcriptomic changes, attributing many apparent DEGs, particularly those related to metabolism and inflammation, to shifts in cell abundance rather than direct transcriptional regulation. Importantly, the correction also unveiled previously masked biological processes associated with the cmAKO-specific response to MI, including pathways related to cell adhesion, cell cycle regulation, and stress response, highlighting potential intrinsic mechanisms of α1A-AR cardioprotection. RNAscope validation supported the composition-aware findings for key genes. This work presents a robust method for dissecting bulk RNA-seq data from complex tissues and provides refined insights into the cellular and molecular roles of cardiomyocyte α1A-ARs during cardiac injury and remodeling.

## Author summary

Traditional methods for exploring changes in gene expression often overlook the fact that tissues are made up of many different cell types, leading to errors in identifying the underlying causes of expression changes. This is particularly common during heart disease which often involves loss of muscle cells, increase in non-muscle cells like fibroblasts, and other changes to cellular composition. To address this, we developed a computational approach to estimate the proportion of various cell types in the heart, helping us distinguish between changes in gene activity and changes in cell composition. Our article focuses on α1A-adrenergic receptors (α1A-ARs), proteins found on the surface of heart muscle cells. α1A-ARs protect the heart during stress but the underlying mechanisms are not fully understood. To study this, we measured changes in gene activity between normal mice and those with a genetic deletion of α1A-AR both in healthy hearts and during heart failure. Our method revealed that much of the apparent change in gene activity during heart injury is due to shifts in cell populations, suggesting that α1A-ARs elicit their cardioprotective effects, in part, by reducing cellular remodeling. This technique may also be applied to numerous other complex diseases which involve changes in cellular composition.

## Introduction

The endogenous catecholamines epinephrine and norepinephrine activate two classes of adrenergic receptors (ARs) in the heart, β-ARs and α1-ARs. Chronic hyperstimulation of cardiomyocyte β1-ARs contributes to the pathobiology of heart failure [1]. In contrast, we and others have shown that α1-AR activation protects cardiomyocytes against multiple forms of injury both *in vitro* and *in vivo* [2,3]. α1-ARs exist as three molecular subtypes: α1A, α1B and α1D. Each subtype is activated by epinephrine and norepinephrine but exhibits distinct tissue localization and cellular signaling. Within the rodent and human heart, the α1A and α1B subtypes are expressed on cardiomyocytes [4,5] whereas the α1D-AR subtype is largely found in coronary artery smooth muscle [6,7].

A burgeoning body of evidence indicates that the α1A subtype mediates the cardioprotective effects of non-selective α1-AR activation [8]. To test whether these adaptive effects require activation of cardiomyocyte α1As, we recently created a cardiomyocyte-specific α1A-knockout mouse line (*Myh6-Cre*x*Adra1a*^fl/fl^ or cmAKO). The cmAKO mice have no discernible basal phenotype but exhibit early mortality and exaggerated pathological ventricular remodeling after myocardial infarction (MI) driven at least in part by unrestrained necroptosis in cmAKO hearts [9]. However, definitive interpretation of these changes by common transcriptomic methods, such as bulk RNA sequencing, is complicated by the fact that MI induces numerous significant changes in the cellular composition of the post-infarct heart including contemporaneous attrition of some cell types and proliferation or infiltration of others. These dramatic changes yield a cellular landscape that is highly heterogeneous, making it difficult to discern whether an observed change in gene expression in bulk tissue is due to changes in the proportional abundance of the cell type(s) that express the gene, altered regulation of the gene itself, or, most likely, a combination of the two. In this setting, where both cell-specific gene expression and cellular abundance are changing, it is a substantial challenge to even ascertain which cell type(s) are expressing a differentially expressed gene and/or driving pathway activation, significantly limiting potential inferences about underlying mechanisms [10–12].

Other studies have frequently used single-cell and -nucleus RNA sequencing to evaluate heterogeneous tissues, but these approaches have severe limitations when applied to our specific experimental design examining cardiomyocyte-specific genetic modifications in the post-MI heart. Beyond the general limitations of cardiomyocyte size (>100 μm) [13] exceeding microfluidic platform capacity [14] and stress-induced multinuclearity of cardiomyocytes [15,16], the cellular response of cmAKO mice to MI introduces physical and experimental complexities that would be difficult to address with single-cell approaches. Studies have shown that the cell-dissociation methods used for scRNAseq often bias the representation of different cell types [15,16] and we expect that this would be particularly relevant in the post-infarct heart, where matrix-embedded fibroblasts and injured cardiomyocytes would be selectively lost. Such biases would particularly impact our ability to measure the differential cardiomyocyte survival that is central to understanding the cardioprotective effects of α1A-ARs. With cells bearing our genetic modification being the most sequencing-challenged major cell type, the very question we seek to answer would be systematically biased in a single-cell experiment. In contrast, bulk RNA sequencing preserves the cellular makeup of whole tissue but masks the specific contributions of each cell type to the overall expression profile [17]. However, the strengths of each approach (cell-type specific expression in single nucleus RNAseq and intact global transcriptomic measures from bulk RNAseq) can be leveraged in reference-based deconvolution while minimizing their respective weaknesses [18].

In this article, we describe a novel method of computationally estimating cellular proportions from bulk RNA sequencing of cardiac tissue. Our approach uses cell-type-specific expression markers to infer the cellular makeup underlying bulk expression from heterogeneous tissue samples, offering a novel means of parsing out the transcriptomic and cellular response of remodeled cardiac tissue. We first identify highly specific cell-type gene expression markers from a snRNAseq reference panel. Then, we validate our analysis pipeline by applying those markers in the deconvolution of pure cell-type ground truth datasets, before estimating cellular abundances from bulk RNAseq from the left ventricles of WT and cmAKO mice, after either left coronary artery (LCA) ligation or sham surgery. We find more exaggerated changes in cardiomyocyte, fibroblast, and immune cell populations in the cmAKO cohort, consistent with a broadly cardioprotective effect of cardiomyocyte α1A-ARs. We simulate the effect of including cell type proportions in differential gene expression analysis of compositionally distinct bulk RNAseq replicates then apply this method of accounting for cellular composition to characterize the transcriptomic changes between our genotype and treatment groups. We find that many expression changes originally attributed to treatment or genotype are better explained by shifts in cellular proportions. Finally, we use RNAscope with IHC to experimentally validate several genes whose significance was altered after adjustment, confirming that several of the most significantly observed transcriptomic changes are accompanied by cell-type-of-origin proliferation or loss. Requiring only pre-existing data types and no additional experimentation, our method offers a convenient, flexible, and affordable approach to simultaneously characterize the cellular state and abundance changes between compositionally distinct groups and has broad applications beyond cardiac data.

## Results

### Unadjusted transcriptional changes in ischemic border zone of cmAKO mice

**Fig 1:**
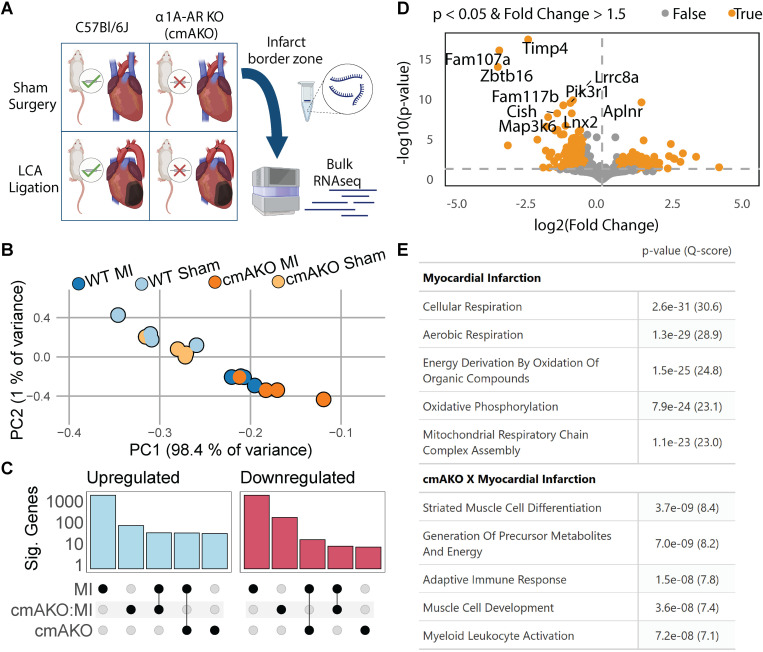
Transcriptomic profiling of cardiomyocyte-specific α1A-AR knockout mice following myocardial infarction (MI). **(A)** Schematic of the experimental design for inducing MI in cmAKO or WT mice and subsequent bulk RNAseq analysis from tissue collected at the infarct border zone and matched sham locations. Figure created in Biorender. **(B)** Principal Component Analysis (PCA) of RNA counts showing stratification of samples by treatment along PC1, accounting for 90% of the variance. **(C)** Differential expression analysis indicating MI as the condition with the highest number of differentially expressed genes (DEGs), followed by the cmAKO and MI interaction; upregulated genes are shown in blue and downregulated genes in red. **(D)** Volcano plot of DEGs specific to the cmAKO response to LCA ligation, highlighting genes like Timp4 (fibroblasts) and Aplnr (cardiomyocytes) with p < 0.05 and fold change > 1.5. **(E)** Gene Ontology (GO) enrichment analysis showing biological processes significantly influenced by MI, cmAKO, and their interaction, such as extracellular matrix organization and cardiac muscle cell development. Terms with identical p-values have been consolidated.

To examine the role of cardiomyocyte α1A-ARs after heart injury, we created a cardiomyocyte-specific α1A-AR knockout mouse line (*Myh6-Cre*x*Adra1a*^fl/fl^ or cmAKO). We induced a myocardial infarction (MI) in this model by permanent LCA ligation at 8–12 weeks of age. In the same mice as described previously [9] we collected tissue from the border zone of the infarcted left ventricle, or a matched location in the sham surgery group, three days post-infarction and measured bulk gene expression by RNAseq (Fig 1A). To identify outliers and unexpected sources of variation that might bias our results, we performed principal component analysis. Samples were well stratified by treatment across PC1, which accounted for 98.4% of the variation in gene expression (Fig 1B). We proceeded with differential expression analysis using DESeq2 [19], modeling gene expression as the sum of the effects of genotype, treatment, and their interaction. LCA ligation produced the greatest number of differentially expressed genes (DEGs), followed by the interaction of cmAKO and LCA ligation, whereas cmAKO alone yielded the fewest DEGs (Fig 1C). These findings recapitulate our prior studies, where we found that α1A-knockout magnified the adverse effects of LCA ligation but elicited minimal phenotypic differences from WT mice in the absence of cardiac insult [9].

When examining the genes that are differentially expressed in the cmAKO × myocardial infarction interaction term (the unique response of cmAKO to LCA ligation), we observe a large number of genes that are enriched in specific cell types, such as Timp4 (fibroblasts) [20], *Zbtb16* (cardiomyocytes) [21], and *Aplnr* (cardiomyocytes) [22] (Fig 1D and S1 Table). Furthermore, gene set enrichment analysis (GSEA) of all interaction-associated DEGs using clusterProfiler [23] revealed strong enrichments of cardiomyocyte- and immune-specific pathways, with the first and third most significant pathways being “striated muscle cell differentiation” (Padj = 3.7e-09) and “adaptive immune response” (Padj = 1.5e-08). Likewise, genes associated with LCA ligation were enriched for cardiac fibrosis and CM energy metabolism processes, including “extracellular matrix organization” (Padj = 1.03e-22) and “cellular respiration” (Padj = 7.92e-24) (Fig 1E and S3 Table). Taken together, these suggest that cell-type compositional changes may contribute to bulk expression changes in the heart. Considering the abundance of expression changes related to cell-type-specific pathways and mechanisms, we next sought to estimate sample-specific changes in cellular composition.

### Cell type-specific markers in snRNAseq

**Fig 2:**
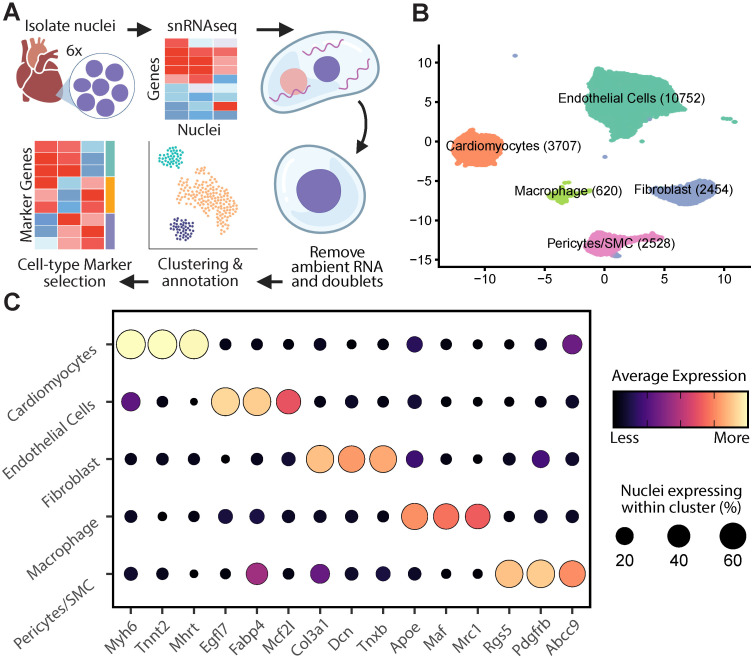
Development and characterization of a cell-type-specific gene expression reference panel from single-nucleus RNA sequencing (snRNAseq). **(A)** Generation of the reference panel involved identifying five major cell types in snRNAseq data from pooled nuclei of left ventricles of untreated C57BL/6J mice. After preprocessing, 15 marker genes per cell type were selected based on significance using the findMarkers function from scran. Figure created in Biorender. **(B)** Annotation and clustering within the snRNAseq reference identified five clusters, each labeled with broad cell type names based on known markers. Post-processing included dimensional reduction and exclusion of clusters with fewer than 400 nuclei or unclear cardiac cell type annotations, resulting in 20,061 nuclei across the five clusters. **(C)** Specificity of cell cluster gene expression markers demonstrated by the top three markers for each cell type, selected by log-fold-change in one-versus-all testing between clusters. Marker visualization includes point size proportional to the nuclei expression and color coding by average expression within each cluster.

To guide the interpretation of our cmAKO model, we developed a cell-type-specific gene expression reference panel. To this end, we performed single nucleus RNAseq from the left ventricles of WT mice (n = 6). Because cell type deconvolution methods have been shown to be sensitive to the specific marker sets and reference dataset used [24], and given the limited application of snRNAseq to deconvolution algorithms, we elected to conduct a rigorous data cleaning and marker selection strategy for our snRNASeq. This began with *in silico* removal of ambient RNA and low-quality nuclei before proceeding with dimensional reduction, and clustering, retaining 20,968 nuclei assigned to 11 clusters (Figs 2A and S1). Next, we sought to connect our nuclei clusters to known cell types by comparing cluster-specific expression markers to known cell-type markers. Cluster markers were identified as genes whose within-cluster expression was significantly higher than out-of-cluster expression in one-versus-all testing using findMarkers from scran [25]. The top 15 most significant marker genes per cluster were supplied to ToppGene to look for enrichment of existing cell type markers. After excluding clusters with less than 500 nuclei or those which presented unclear cell-type associations, the final dataset contained 20,061 nuclei assigned to five cell-type clusters. In order of most-to-least abundant, we retained clusters of endothelial cells, cardiomyocytes, fibroblasts, macrophages, and a joint cluster of pericytes and smooth muscle cells (Fig 2B). Further, we found that our marker list recapitulates standard proteomic and transcriptomic cardiac cell-type markers, including Myh6 [26] and Tnnt2 [27] for cardiomyocytes, Egfl7 [28] and Fabp4 [29] for endothelial cells, and Dcn and Col1a1 [30] for fibroblasts (Fig 2C and S2 Table)

### Cell-type composition of mouse left ventricular tissue

**Fig 3:**
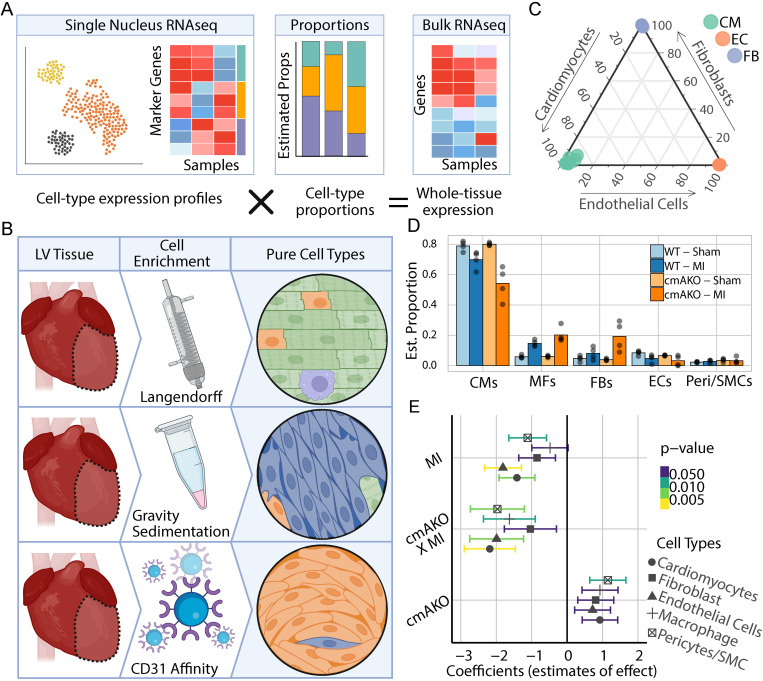
Inference of cell-type proportions from bulk RNAseq and dynamics during myocardial infarction in α1A-AR-KO mice. **(A)** Overview of the reference-based deconvolution approach which utilizes gene expression markers to estimate the proportions of cell types contributing to bulk gene expression. Figure created in Biorender. **(B)** Generation of cell-type enriched bulk RNAseq data involved isolating cardiomyocytes, fibroblasts, and endothelial cells from left ventricles of untreated WT mice using Langendorff perfusion, gravity sedimentation, and CD31-bead binding. Figure created in Biorender. **(C)** Validation of the deconvolution pipeline using bulk RNAseq from pure cell type fractions as ground-truth samples, depicted in a ternary plot where each dot represents a replicate, color-coded by intended enriched cell type, indicating high accuracy of the composition estimates. **(D)** Estimation of cardiac cell type composition changes post-myocardial infarction (MI) and in α1A-AR knockout (cmAKO) mice, showing increased proportions of macrophages and fibroblasts and decreased proportions of cardiomyocytes, with more pronounced changes in cmAKO mice. **(E)** Results from a Dirichlet regression modeling cell type proportion as influenced by cmAKO, MI, and their interaction, revealing significant effects on several cardiac cell types. CMs = Cardiomyocytes; ECs = Endothelial Cells; FBs = Fibroblasts; WT = Wild-Type; MI = Myocardial Infarction.

Cell-type deconvolution is a method to infer how much RNA comes from each cell type present in bulk RNA sequencing data produced from heterogeneous tissue. As such, it is important to note that these approaches do not estimate the number of each type of cell nor their total volumes. For our pipeline, we chose to utilize MuSiC, one of several existing deconvolution methods, which iteratively tests for the combination of cell type proportions whose summed expression profiles best explains the overall tissue expression profile. (Fig 3A). To inform development of our analysis pipeline and to test its performance, we first applied it to several bulk RNAseq datasets derived from purified fractions of major cardiac cell types (Fig 3B). We found that our MuSiC-based method was able to accurately predict the makeup of every cardiomyocyte, endothelial cell, and fibroblast sample (Fig 3C). Confident in our ability to identify major cell types of the heart, we then applied our pipeline to the bulk RNAseq data from our experimental groups. In the untreated WT mice, we estimated that the major cell type *by transcript abundance* was cardiomyocytes (79%), followed by fibroblasts (8.4%) and macrophages (5.8%). For both genotypes, cardiomyocyte abundance decreased in the treatment group, while fibroblasts and macrophages rose in relative abundance. For each of these changes, these compositional shifts were more pronounced in cmAKOs when compared to the WT group (Fig 3D).

Traditional models and association tests, such as the Pearson correlation [31], are prone to identifying spurious correlations when applied to contexts where measures are interrelated, as with cellular composition data [32]. To understand this, imagine a scenario where a treatment causes cardiac fibroblasts to proliferate while having minimal effect on adjacent cell types. If researchers were to quantify the number of each cell type in a treated heart, they would find that the absolute number of non-fibroblasts remains constant. However, since the total number of cells in the heart increased, the relative proportion of non-fibroblasts would seem to decrease compared to an untreated heart. As such, standard statistical tests applied to these compositional measures would report that the treatment is associated with both an increase in fibroblasts and a decrease in non-fibroblasts, despite there being no absolute increase in the number of non-fibroblasts.

To address this and correctly quantify the effect of each treatment and genotype on the compositional changes, we used a Dirichlet regression model, which can accommodate the sum-to-one constraint and covariance structure of compositional data [33,34]. Our model considered the effects of each genotype and treatment additively, as well as the interaction of the two terms. We found that MI and the cmAKO × MI interaction are the only terms to have significant effects on major cell type proportions. Specifically, MI and cmAKO × MI were both significantly associated with the abundance of cardiomyocytes (p = 0.0051 for MI; p = 0.0024 for cmAKO × MI), endothelial cells (p = 0.00053 for MI; p = 0.0089 for cmAKO × MI), and pericytes/SMC (p = 0.036 for MI; p = 0.01 for cmAKO × MI). Additionally, only cmAKO × MI was found to be significantly associated with the proportion of macrophages (p = 0.026) (Fig 3E).

The more pronounced cellular compositional changes in cmAKO mice compared to WT following MI suggest that α1A-ARs play a critical role in maintaining cardiac cellular homeostasis during stress. The significant loss of cardiomyocytes and increase in fibroblasts in α1A-KO + LCA mice, which was attenuated in WT + LCA animals, indicates that α1A-AR signaling may protect cardiomyocytes from stress-induced death and limit the fibrotic response. These findings provide a novel cellular mechanism that may underlie the cardioprotective effects of α1A-ARs previously observed in various models of cardiac injury.

### Cell-type-adjusted differential gene expression

**Fig 4:**
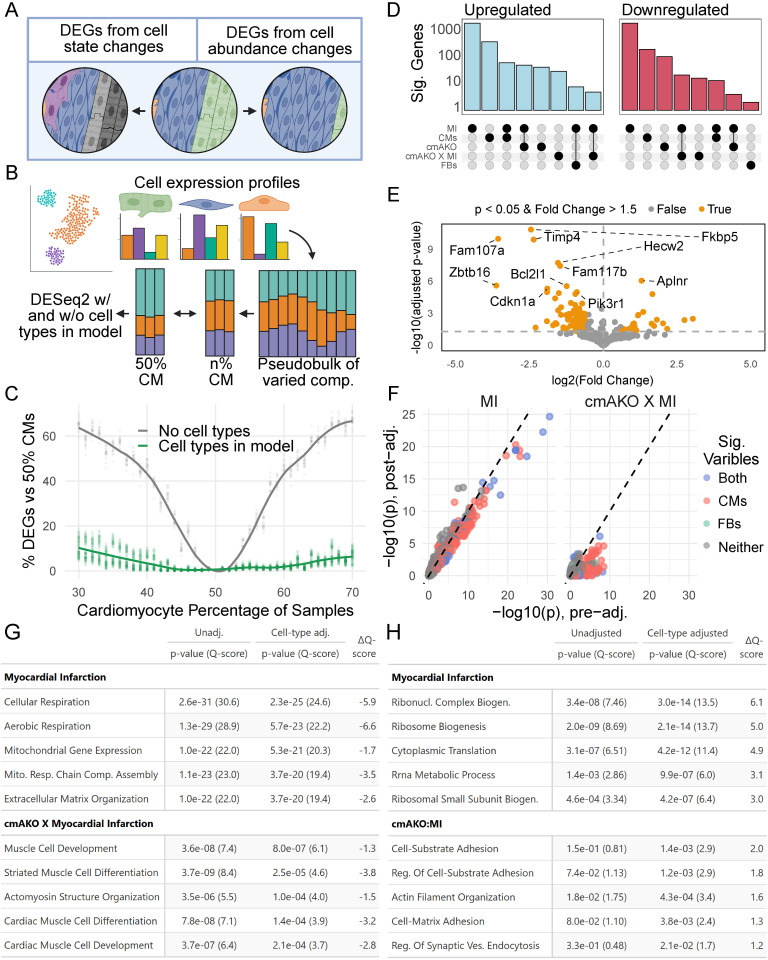
Impact of cell type composition on differential gene expression during myocardial infarction in α1A-AR-KO mice. **(A)** Differences in either cell type abundances or novel states can lead to gene expression differences in bulk tissue. Figure created in Biorender. **(B)** Schematics of one simulation of compositional changes in differential expression; bulk RNAseq samples were generated with varying cardiomyocyte proportions (30-70%). All samples were compared against the 50% CM group with and without cell-types in the model. **(C)** DESeq2 simulations show increases in differentially expressed genes with compositional differences; including cardiomyocyte proportions in the analysis ablates this effect. Each dot is one simulation of 500 genes. **(D)** Upset plot showing DEGs related to each variable after adjusting for cell-type composition. Cardiomyocyte proportion is the second strongest associated variable. **(E)** Volcano plot showing differential expression of genes associated with cmAKO × MI interaction after adjusting for cell type proportions. **(F)** Changes in GO term associations and their significance before and after cell type adjustment, depicted through a scatter plot of Q-scores. Many myocardial infarction-associated GO terms are attributed to changes in cell type abundance. **(G)** and **(H)**, Listing of top GO terms post-adjustment showing significant associations with myocardial infarction and cmAKOxMI **(G)**, and with changes in cardiomyocytes and fibroblasts **(H)**.

Variation in either cell state or abundance [35] can be responsible for differences in the overall transcriptional profiles of cellularly heterogeneous tissue (Fig 4A). Given the differences in cellular abundance in our samples, we became interested in discerning the contribution of these compositional differences to the transcriptional changes we observed in response to LCA ligation and CM-α1A KO. However, directly including cell type proportions as covariates in statistical models can lead to inflated associations since proportions are constrained to sum to one and thus inherently correlated [36,37]. To address this, we evaluated different transformations of the proportions, including principal components analysis and centered log-ratio (CLR), which converts proportions to an unconstrained space while preserving their relationships [38].

To model the inclusion of compositional estimates in gene expression analysis, we simulated bulk RNAseq sample groups composed of a range of underlying cellular proportions (Fig 4B). *In silico* mixtures were generated by sampling expression of individual genes from each cell-type cluster according to pre-specified cell-type ratios. We began with 50% cardiomyocytes and equal proportions of each remaining cell type from our snRNAseq panel. We then introduced stepwise changes in cardiomyocyte proportion from a range of 30–70% cardiomyocytes. Then, for non-control groups, we doubled the expression of 10% of random genes to serve as true positives. We then tested for differential gene expression between the 50% cardiomyocyte group and each other group. In these comparisons, we found a considerable effect of compositional changes on differential expression. Strikingly, a 10% reduction in the major cell type, a change smaller than that seen in cardiomyocyte abundance during MI in our data, caused 20% of transcripts to be identified as differentially expressed genes (DEGs) (Fig 4C).

When we repeated the analysis including different representations of the cell type proportions in the model (raw percentages, principal components analysis (PCA), or centered log-ratio (CLR) transformations), we found that including any of these compositional covariates dramatically improved the accuracy of DEG detection compared to the unadjusted model (S2 Fig), with PCA offering the strongest overall performance. However, given that CLR transformation still provided substantial improvements over the unadjusted model and is an established, is theoretically grounded method for compositional data analysis, and offers more direct biological interpretation related to specific cell types compared to the abstract components derived from PCA, we elected to utilize CLR adjusted models for analyzing our experimental data [38]. Nonetheless, incorporating compositional information via any of these methods clearly shifted the attribution of expression changes away from the sample group comparisons (which were blind to composition) and towards the compositional covariates themselves. These results demonstrate how comparisons of bulk gene expression can be skewed when sample groups have confounding variation in cell composition and how including compositional terms in DEG analysis enables more accurate attribution of expression changes.

Applying our cell abundance correction method significantly impacted the interpretation of gene expression changes, revealing that cardiomyocyte abundance was associated with the expression of hundreds of genes (Fig 4D). While LCA ligation remained the most influential variable overall, accounting for cell-type proportions clarified the source of many transcriptional signals (Fig 4D; S3 Table).

In the comparison between WT MI and WT Sham hearts, correction attributed a substantial portion of the initial differential expression signature to changes in cellular composition. Hundreds of genes exhibited reduced significance, with many losing statistical significance altogether after adjustment (S3 Table). For instance, the apparent strong upregulation of the epicardial marker *Upk3b* [39] (Padj = 4e-42 to 8e-22) and several complement cascade genes like *Cfb* and *C4b* (approx. 20 orders of magnitude reduction) in the uncorrected analysis likely reflected shifts in cell abundance and inflammatory cell infiltration, respectively, rather than direct transcriptional regulation within resident cell types due to infarction. This reattribution was mirrored in GSEA findings, where the association between MI and terms related to cardiomyocyte energy metabolism (e.g., “oxidative phosphorylation,” Padj = 7.92e-24 to 3.29e-19; “generation of precursor metabolites and energy,” Padj = 7.26e-19 to 3.27e-13) weakened considerably, suggesting these changes were largely driven by the loss of cardiomyocyte populations (Fig 4F, G; S4 Table).

Conversely, the correction unveiled a smaller, yet biologically insightful, set of genes whose association with MI strengthened (S1, S3 Tables). Notably, *Lgals3*, previously implicated in heart failure and the post-MI remodeling response [40–43], became highly significant only after correction (Padj = 0.16 to 1e-7), highlighting it as a potential candidate for future investigation. These findings aligned with GSEA results showing strengthened associations for terms related to protein synthesis (e.g., “ribonucleoprotein complex biogenesis,” Padj = 3.45e-8 to 3.04e-14; “cytoplasmic translation,” Padj = 3.08e-7 to 4.25e-12), consistent with recent novel evidence of altered translational activity in cardiomyocytes post-MI [44] (Fig 4H)

Within the cmAKO × MI interaction contrast, fewer genes were impacted by the correction compared to the main MI effect, but the adjustment still refined the interpretation (S3 Table). Key genes initially strongly associated with the interaction, such as the cell cycle regulator *Zbtb16* (Padj = 9e-15 to 2e-6) and the inflammatory modulator *Timp4* (Padj = 4e-18 to 1e-10), showed reduced significance. Given the high expression specificity of these genes in cardiomyocytes [21] and cardiac fibroblasts [20] respectively, this suggests their apparent genotype-specific response was largely influenced by differences in cell composition shifts (e.g., cardiomyocyte loss or fibroblast/immune infiltration) between WT and cmAKO mice post-MI. Accordingly, GSEA terms related to energy metabolism and immune activation (e.g., “response to bacterium,” “generation of precursor metabolites and energy”) weakened in their association with the cmAKO × MI interaction after correction (S4 Table).

Notably, the composition-adjusted analysis revealed strengthened associations between the cmAKO × MI interaction and processes pertaining to cellular stress, structure, and metabolism (Fig 4G, H). GSEA highlighted pathways related to cell adhesion (“actin filament organization,” Padj = 1.77e-02 to 4.29e-04; “cell-substrate adhesion,” Padj = 0.155 to 1.38e-03) and cell cycle regulation/stress response (“G1/S transition of mitotic cell cycle,” Padj = 0.293 to 2.9e-02; “DNA damage response via p53 mediator,” Padj = 0.435 to 4.11e-02), several of which became significant only after correction. Individual genes driving these enrichments included *Cdkn1a*, a known regulator of cardiomyocyte cell cycle [45,46] implicated in heart failure [47], which increased in significance (Padj = 6.04e-03 to 4.7e-06). *Tent5b*, involved in apoptosis and glycolysis [48] relevant to the cmAKO metabolic phenotype, also became significant (Padj = 0.38 to 3.71e-04). Similarly, *Fhl1*, linked to cardiomyopathy and muscle metabolism [49,50], gained significance (Padj = 0.477 to 5.27e-04), potentially connecting α1A-AR’s anti-glycolytic function [51] to *Fhl1* activity. These newly prominent genes and processes suggest that α1A-ARs may exert cardioprotective effects by influencing cytoskeletal integrity, cell cycle control, and stress response mechanisms—functions obscured in the uncorrected analysis by confounding compositional changes (S3 Table).

Collectively, these results highlight the critical importance of accounting for cell-type composition in bulk RNA-seq analysis (Fig 4). By distinguishing genuine transcriptional responses within cell types from changes arising due to altered cellular abundances, our method refines the interpretation of the myocardial infarction response, particularly in the context of the cmAKO model. Genes whose significance increased after correction represent potentially crucial intrinsic regulatory mechanisms underlying α1-adrenergic cardio protection, while those losing significance reflect secondary population shifts. Thus, our composition-aware approach provides a more biologically accurate understanding of myocardial transcriptional responses to injury.

### Spatial expression of cell type and cell state markers

**Fig 5:**
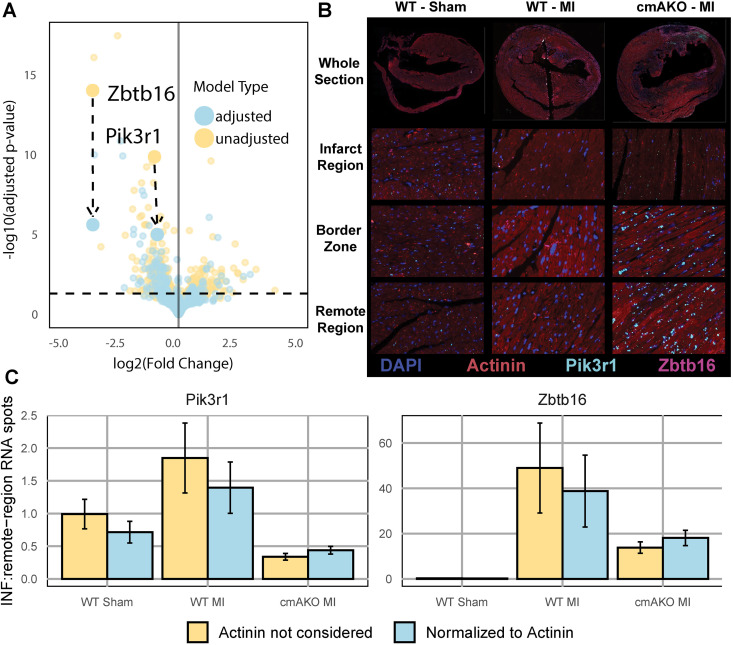
Fluorescent in-situ hybridization experimentally validates composition-aware differential gene expression results. **(A)** Volcano plot showing expression changes associated with the unique response of cmAKO mice to myocardial infarction before (yellow) and after (blue) including terms for cardiomyocyte and fibroblast abundance in the DESeq2 model. Two highly significant genes, Zbtb16 and Pik3r1, are representative of a pattern of reduced significance in the updated model. **(B)** RNAscope of Pik3r1 (cyan) and Zbtb16 (purple) overlayed with IHC showing DAPI (blue) and Actinin (red) in mouse left ventricular tissue. Representative images of the border zone, infract region, and a remote region from sham surgery control wild-type mice, wild-type mice after LCA ligation and cmAKO mice after LCA ligation. **(C)** Adjusting for cardiomyocyte abundance minimizes differences in spatial expression of Pik3r1 and Zbtb16 in the infarct region, indicated by the post-adjustment expression increases in cmAKO MI and reductions in WT MI. Five representative regions were evaluated in each zone from (B) and the number of expression spots were tallied for each gene. Actinin was used as a proxy for cardiomyocyte abundance and spot counts were normalized to actinin abundance (blue) or not (yellow). To control inter-slide technical variation, spot counts were normalized to slide-matched measures in a remote region (shown on the y-axis).

To validate the ability of our model to predict regulation of transcripts within distinct cell types, we used RNAscope paired with immunohistochemistry. We designed probes for *Zbtb16* and *Pik3r1,* two highly regulated transcripts in the bulk RNAseq data that experienced large reductions in significance in their association with the cmAKO interaction with MI upon adjusting for shifts in cellular composition (Fig 5A). We then localized those probes within cardiomyocytes using immunofluorescent staining for sarcomeric alpha-actinin as an additional control for our methods.

Our predictive model indicated that *Zbtb16* (a zinc finger transcription factor), and *Pik3r1* (PI 3-kinase regulatory subunit 1) were regulated in a genotype-specific manner within cardiomyocytes after MI. Both transcripts are known to be expressed in cardiomyocytes [21,52], but no previous studies have identified them as downstream targets of α1-ARs. We found that both *Zbtb16* and *Pik3r1* were expressed in cardiomyocytes under all conditions. Control hearts (Fig 5B) displayed uniform transcript expression across tissue regions. Consistent with our predictions, transcript abundance of cardiomyocyte *Zbtb16* was low in ligated WT mouse hearts compared to the robust upregulation in hearts from ligated cmAKO mice. The pattern of *Pik3r1* expression was identical, although the magnitude of differential expression was qualitatively lower, also consistent with our predictions.

To quantify these effects, we evaluated five representative subregions within the border zone, infarct region, and remote region (or matched locations) of each slide. For each subregion, we tallied the number of expression spots of each gene, as well as the area occupied by actinin, which was used as a proxy for total cardiomyocyte cross-sectional area. To account for differences in cardiomyocyte abundance (S3 Fig), we then normalized expression spot counts to the area occupied by actinin in each subregion. Due to the technical variation present between each image, we further normalized these values to those from the remote region of each sample. We then observed that accounting for cardiomyocyte abundance reduced the differences in spatial expression both *Zbtb16* and *Pik3r1* between cmAKO and WT mice during myocardial infarction (Fig 5C). This finding is consistent with the predictions made in the composition-aware differential expression analysis, which suggested that *Zbtb16* and *Pik3r1* expression in bulk tissue was affected by cell type proportion shifts. See S6 Table for full results of quantification and parameters needed to reproduce them.

## Discussion

Changes in cardiac cell composition that occur during cardiac remodeling are a hallmark of cardiac dysfunction. Notably, loss of cardiomyocytes and proliferation of fibroblasts has been previously reported in myocardial infarction, trans-aortic constriction, and beta-adrenergic overdrive models of heart disease [53,54]. Quantitative analyses of the degree of remodeling in the heart remains complicated due to a variety of technical and biological factors. In this study, we have developed a computational method that accounts for transcriptomic changes in bulk RNA sequencing data by delineating cell-type specific changes. We explore the efficacy of this approach in bulk RNAseq datasets drawn from wild type and cardiomyocyte-specific α1A-AR knockout mice (CM-α1A-KO) subjected to myocardial infarction or sham surgery.

While significant advances have been made in single-cell technologies, these approaches remain particularly ill-suited for the experimental design we employed. Our experimental design (genotype × treatment) generates a complex cellular landscape with interaction effects at both the cellular composition and gene expression levels. Technical limitations of single-cell approaches are magnified in this context: the extensive fibrosis in post-MI tissue creates substantial cell isolation bias; cardiomyocytes show variable multinuclearity in mice, are too large for standard microfluidic capture; and the relative cost limits powered comparisons across the four experimental groups. The statistical efficiency of bulk RNA-seq with computational deconvolution proves advantageous for designs like ours where cellular composition changes are a key aspect of the biological response. Rather than attempting to directly measure thousands of individual cells with uncertain representativity, our approach leverages prior knowledge from reference snRNA-seq data to enable accurate inference of composition changes from statistically powerful bulk measurements.

A growing body of literature demonstrates that α1A-ARs play adaptive and protective roles in cell culture and animal models [2,55]. We recently found that selective antagonists of α1A-ARs such as tamsulosin (Flomax), used commonly to treat lower urinary tract symptoms related to benign prostatic hypertrophy, are associated with a small but statistically significant increase in one-year mortality in a subset of the Medicare database [56]. To understand the primacy of cardiomyocyte α1A-ARs in these findings, we created a mouse line (cmAKO) with cardiomyocyte-specific deletion of the α1A-AR and found that cmAKO mice had markedly increased mortality and exacerbated pathological ventricular remodeling following myocardial infarction (MI) [9].

Applying our computational deconvolution algorithm to this model enabled us to pinpoint how shifts in cell populations, like increases in fibroblasts and decreases in cardiomyocytes, directly influence gene expression changes during cardiac injury. Strikingly, we observed that cardiomyocyte abundance presented the strongest associations with biological processes, reflecting the outsized effects of cardiac cell composition on differential gene expression. Many of the most significantly differentially expressed genes in conventional analysis resulted from combinations of shifts in cell composition and intrinsic gene expression that act to magnify their effect. Importantly, our novel computational approach facilitated the identification of novel areas for future mechanistic study that could expand our understanding of myocardial alpha-1-adrenergic receptors. GSEA analysis of DEGs in our deconvoluted dataset identified three pathways that were enriched in the cmAKO x myocardial infarction dataset but were not statistically significant in the GSEA analysis of the original bulk RNAseq dataset: Cell-substrate adhesion, Regulation of cell-substrate adhesion, and cell-matrix adhesion (S4 Table). These pathways are quite relevant to the context, as interactions between numerous cardiac cell types and the extracellular matrix are critical to the pathobiology of the infarcted heart (reviewed [57] in [57–60]). However, there is no published role for alpha-1-adrenergic receptors in regulating these critical processes.

Our analysis also pinpointed novel targets within the cmAKO × MI interaction, aligning with corrected GSEA terms related to cell adhesion, cell cycle, and stress response pathways. Notably, genes such as *Cdkn1a*, involved in cell cycle regulation and senescence [61,62], and *Fhl1*, linked to biomechanical stress and altered metabolism [49,63], gained significance only after accounting for cell composition (S3 Table). The non-canonical poly(A) polymerase *Tent5b* [64], involved in RNA metabolism, also emerged as significant in this context. *Cdkn1a* and *Fhl1* are particularly interesting targets as their contributions to the pathobiology of cardiac stress have been studied extensively in the preclinical setting [65–68] and neither have yet been associated with α1A-ARs.

While several methods to account for cell composition in bulk RNAseq exist (including TOAST [69], BMIND [70], and CARseq [71]), these approaches have important limitations in the context of our study. To our knowledge, no cell-type-specific gene expression inference tool has been developed for or benchmarked in the cardiac context, as is the case for the broader field of cell type deconvolution. In the only benchmarking study of these methods to-date, the authors found that the accuracy of cell type-specific differential expression detection is impaired by the very factors that define post-MI remodeling: large shifts in cell composition and the need to detect signals in low-expression genes [72]. For instance, BMIND is noted by its authors to be less sensitive for less abundant cell types, a limitation when studying fibroblast proliferation or cardiomyocyte death in highly heterogenous tissue regions. TOAST, while effective for improving feature selection, was designed primarily for microarray data and to assist reference-free deconvolution, which is generally less accurate than the reference-based approaches used in our study. Additionally, benchmarking has only been performed by including raw proportions as covariates in differential expression model [72], an approach that violates statistical assumptions due to the compositional nature of the data. Our study uniquely addresses these limitations by systematically leveraging cell type estimates through centered log-ratio transformations and conducting internal benchmarking with simulations specifically designed to mimic cardiac remodeling scenarios.

This workflow provides a distinct practical advantage; by building upon DESeq2, one of the most widely used and actively maintained tools for transcriptomics, our method is both easily adoptable for researchers and more flexible than relying on bespoke packages that may not receive continued support. Therefore, we position our method as an alternative to, rather than complete replacement, for existing tools, with the most reliable hypothesis generation likely to come from results which align across multiple methods. This allows researchers to leverage the statistical power, comprehensive transcriptome coverage, and cost-effectiveness of bulk RNA-seq while providing biologically interpretable results about both cellular composition and state changes during complex cardiac remodeling.

The practical advantages of our approach are especially relevant for experimental designs like ours. Alternative spatial or single-cell based approaches face substantial barriers: spatial transcriptomics methods like Slide-seq have lower gene detection sensitivity [73] and significantly higher costs and combinatorial barcoding approaches for single cell require specialized expertise [74] unavailable to most labs. In contrast, our computational approach allows researchers to leverage the statistical power, comprehensive transcriptome coverage, and cost-effectiveness of bulk RNAseq while still accounting for the cellular heterogeneity inherent in complex cardiac disease models. This is particularly valuable for cardiac studies involving post-MI tissue, where the cellular remodeling itself is a central aspect of the biological response to be measured, not merely a confounder to be controlled.

Researchers have produced a tremendous amount of bulk RNAseq data in the last decade, and it remains a first-line tool for profiling transcriptomes. More than 11,000 bulk RNA-seq datasets have been deposited in NCBI’s Gene Expression Omnibus (GEO) and 79% of NGS data deposited in 2022 was from bulk RNA datasets, rather than single-cell studies [75]. As such, this approach opens new avenues for both retrospective and prospective research projects. Existing cohorts may be reanalyzed, potentially uncovering novel insights that were previously obscured due to changes in cellular abundance. Furthermore, by correcting for cell-type abundances, our method helps to refine models of disease mechanisms by delineating effects to their cell-type of origin, thereby improving the predictive accuracy of transcriptomic markers of disease.

While our computational method significantly enhances bulk RNA sequencing analysis from heterogeneous samples, it is not without limitations. One key challenge is the reliance on accurate cell-type-specific markers. Incorrect or suboptimal marker selection can lead to inaccurate deconvolution results, which may obscure true cellular contributions to gene expression changes. Our method is also constrained by the cell types and states present in the supplied reference, which may require researchers to generate their own references for specific biological contexts. If cell types with expected proportion changes are excluded from the study, their effects may be misattributed to other, highly correlated cell types. Additionally, while our method improves the resolution of cellular contributions, the broader field of cell type deconvolution struggles to maintain accuracy as the number of included cell types and/or their similarity to one another increases [76]. Further, this method estimates the relative proportions of RNA from each cell type, not their total numbers or size. While this is preferred for our use-case (adjusting bulk transcriptomics) connecting experimental variables to cell volume or number may be more optimal for other research contexts. Future efforts could further improve this approach by integrating spatial transcriptomic validation, developing more specific markers for closely related cell states, and benchmarking deconvolution algorithm performance across different tissue contexts and disease states. The growing availability of comprehensive single-cell atlases will enable increasingly accurate reference development for deconvolution approaches.

In this study, we defined the transcriptomic signatures that result from the absence of cardiomyocyte α1A-ARs in the uninjured and post-infarct state. Our experimental design examines both genotype and treatment effects simultaneously, creating the ideal scenario to demonstrate the value of composition-aware analysis, as both variables independently alter cellular makeup in complex ways. Leveraging a novel computational approach, we accurately estimated cell type-specific contributions within bulk RNA sequencing data, overcoming the technical barriers that would have made single-cell approaches impractical for this experimental design due to cell type sampling bias and cost. Further, we validated the method by visualizing the spatial expression of two representative transcripts, recapitulating the findings from our computational model. This enabled us to precisely dissect how shifts in cellular composition directly impact gene expression during cardiac stress, revealing that α1A-ARs elicit their cardioprotective effects partly by reducing cellular remodeling. The resulting composition-aware dataset identified novel associations with pathways and individual transcripts that will inform future mechanistic studies to expand our understanding of the cardioprotective effects of α1A-ARs. This study not only contributes to our understanding of the molecular dynamics within the heart but also offers a robust, scalable strategy to uncover hidden insights in complex experimental designs where cellular heterogeneity is a central feature of the biological response.

## Materials and methods

### Ethics statement

All experiments were approved by the IACUC review boards of their respective institutions and mice housed in AAALAC-accredited vivariums. The specific relevant review boards are University of North Carolina at Chapel Hill Institutional Animal Care and Use Committee (IACUC), the UCLA Chancellor’s Animal Research Committee, and the Medical College of Wisconsin Institutional Animal Care and Use Committee.

### Mouse husbandry and model generation

cmAKO mice: C57BL/6J and ROSAmT/mG (stock # 007676) mice were purchased from Jackson lab. The cmAKO mouse line was generated by breeding Myh6-Cre (originally from the lab of E. Dale Abel at the University of Iowa and provided by Leslie Leinwand at the University of Colorado) to Adra1a flox/flox mice with loxP sites flanking the first coding exon (constructed in the Paul C. Simpson lab at the University of California, San Francisco/San Francisco VA Medical Center) [77,78]. All mice were backcrossed regularly and maintained on a C57BL/6 genetic background. Twelve- to 16-week-old males were used to generate the myocardial infarction and sham-operated mouse models. Floxed mice (αMHC-Creneg/α1Afl/fl) were used as wild-type (WT) controls for cmAKO mice.

snRNAseq experiments: C57BL/6J mice (JAX stock 000664) were purchased from Jackson Laboratory, Bar Harbor, Maine, and bred to produce pups.

Pure Cell fractions: For experiments involving pure cell-type fractions, C57BL/6J mice were obtained from Jackson Laboratories at 7 weeks of age and allowed to acclimatize for at least two weeks.

### Myocardial infarction model

Mice were subjected to permanent LCA ligation as previously described [9]. In brief, to induce myocardial infarction (MI), a left thoracotomy was performed at the fourth-fifth intercostal space, followed by permanent ligation of the left anterior descending (LAD) coronary using a 7/0 non-absorbable ethylene suture. Occlusion was verified by anemia and akinesis of the apex and anterior-lateral wall, after which the thorax was closed in layers. After extubation, mice were kept warm until fully recovered. For LCA ligation, sham surgery, and terminal cardiectomy, mice were anesthetized by inhalation of isoflurane (2%). For postoperative analgesia, 5 mg meloxicam/kg body weight was applied every 24 hours for the first 72 hours post-surgery.

### Tissue collection and preparation

After sacrifice, the heart was quickly excised and sectioned perpendicular to the long axis of the left ventricle. The infarct border zone was visualized under a dissecting microscope and dissected out then immediately snap frozen in liquid nitrogen. Tissue was taken from an anatomically analogous location in sham-operated hearts using an identical process. RNA was extracted and shipped to Novogene (Sacramento, CA) for bulk RNAseq as described below.

To fix heart tissue for immunohistochemistry, mice were heparinized, and the heart was perfused with 10 mL of PBS followed by 20 mL of 4% paraformaldehyde (PFA)–PBS through a 23-gauge butterfly needle, then excised and placed in 4% PFA-PBS for 24 hours before transfer to 70% ethanol. Hearts were then embedded in paraffin and 5 µm sections collected.

### Fluorescent In*-Situ* Hybridization (FISH)

Formalin-fixed paraffin sections were used for localizing the cellular expression of various mRNA transcripts by *in situ* hybridization with the RNAscope Assay [79] as described by the manufacturer (Advanced Cell Diagnostics, Inc., Newark, CA). Briefly, slides were baked at 60°C for 1 h, deparaffinized with xylene and absolute ethanol, and pretreated with Target Retrieval Reagent, H_2_O_2_, and Protease Plus according to manufacturer specified conditions and times. This was followed by hybridization with RNAscope Probes for 2 h at 40°C in the HybEZ oven for detection of mRNA, or a negative control probe (S5 Table). RNAscope Multiplex Fluorescent Reagent Kit v2 (Advanced Cell Diagnostics, Inc., Newark, CA, Cat No. 323120) was employed for signal amplification and detection. This kit uses fluorescent probes for the development of a reaction product visible at the Cy5 and Cy7 channels. FITC was not used due to green channel autofluorescence common in the heart.

### Immunofluorescence staining and image acquisition

Immediately following FISH, slides were blocked with 10% normal goat serum for 1 h and incubated with primary antibody overnight at 4°C (S5 Table) Next, slides were incubated with secondary antibody for 2 hr at RT. Slides were then rinsed in PBS and mounting medium with DAPI was applied and coverslips were attached and sealed with clear nail polish. FISH and IF staining was performed in the University of North Carolina Histology Research Core.

Stained sections were imaged with an Olympus VS200 slide scanner equipped with a motorized stage, an Olympus DP74 digital camera, and OlyVIA software (Olympus America Inc., Center Valley, PA, RRID: SCR_016167). These full scans were opened using Qupath (RRID: SCR_018257), and single field images were generated [80]. (Raw data at: https://doi.org/10.17632/bskhbpf9pp.1)

### Image analysis and quantification

Quantification of RNAscope fluorescent in-situ hybridization (FISH) spots and immunohistochemistry (IHC) staining area was performed on single-field images generated from full slide scans using QuPath (v0.5.1) [80]. For each slide, five representative, non-overlapping 300x300 pixel regions of interest (ROIs) were manually selected within the infarct border zone, infarct region, and remote region (or anatomically matched locations in sham controls).

Within each ROI, automated spot detection was used in QuPath to count the number of FISH spots for *Zbtb16* (Cy5 channel) and *Pik3r1* (Cy7 channel). The total area (in pixels) positive for sarcomeric alpha-actinin IHC staining (FITC channel) was measured using intensity thresholding to serve as a proxy for cardiomyocyte area within the ROI.

To account for variations in cardiomyocyte density, the spot count for each gene (*Zbtb16*, *Pik3r1*) in each ROI was normalized by dividing it by the corresponding actinin-positive area within that same ROI, yielding a spot density value. To control inter-slide technical variability, the spot density value for each border zone and infarct region ROI was further normalized by dividing it by the average spot density calculated from the five remote region ROIs on the same slide. The final normalized values are presented in Fig 5C, and the raw quantifications and parameters needed to reproduce this analysis for each ROI are available in S6 Table.

### Cardiac cell isolation for pure cell type fractions

As previously described, adult B6 mice were treated with heparin (100 USP units) for 20 minutes to inhibit blood coagulation followed by anesthesia with sodium pentobarbital (100 µL of 50 mg/ml dilution, intraperitoneal) [81]. Upon loss of rear foot reflex, hearts were removed and immediately submerged in ice-cold PBS to arrest the heart.

#### Cardiomyocytes.

Hearts were mounted on a modified Langendorff apparatus and perfused for 5 minutes with Tyrode’s solution (10u mM NaCl, 5.4 mM KCl, 1 mM MgCl2, 0.6 mM Na2HPO4, 10 mM glucose, 10 mM HEPES (pH 7.37), oxygenated with 95% (v/v) O2 and 5% (v/v) CO2) at 37°C, then perfused for 15–30 minutes with 30 mL Tyrode’s containing 20 mg collagenase type-II and 3mg protease type-XIV, then washed for an additional ten minutes with Krebs buffer (25 mM KCl, 10 mM KH_2_PO_4_, 2 mM MgSO_4_, 20 mM glucose, 20 mM taurine, 5 mM creatinine, 100 mM potassium glutamate, 10 mM aspartic acid, 0.5 mM EGTA, 5 mM HEPES (pH 7.18) oxygenated with 95% O2 and 5% CO2 (v/v)). Cardiomyocytes were then dissociated in Krebs buffer, filtered through a 100 µm strainer, and centrifuged 2 minutes at 1000G before being placed in triZoL for RNA isolation.

#### Fibroblasts.

Fibroblasts were isolated through enzymatic digestion using Liberase (Roche, 5401119001). Hearts were minced into small pieces and transferred into 18 mL of 1x Liberase in Hanks (+Ca, + Mg) media. Solution is gently stirred while incubating at 37°C for 3 minutes. Tissue is allowed to settle, then the supernatant is sieved through a 70 µm strainer and transferred to a tube containing 10 mL of Krebs-Henseleit (KH) buffer (Sigma, K3753). Cell suspension is centrifuged at 1000 rpm for 5 minutes in a (Sorvall Legend Micro 17 Centrifuge). Supernatant was removed and pellet washed with 10 mL ice-cold KH buffer, followed by another round of centrifugation and resuspension in 5 mL of cold KH buffer. The process was repeated using the rest of the heart tissue 4 times until no clumps of heart tissue remained in the original tube. All cells were centrifuged then plated on untreated plates in DMEM/F12 media supplemented with 10% FBS, 1% Pen/Strep and 0.1% insulin-transferrin-selenium (ITS; Corning, 354350). After 2 h, human basic fibroblast growth factor (1:10,000 concentration from a 200x stock, MilliporeSigma, 11123149001) was added to the media. Media was removed after 24 h, plates were washed with PBS, then triZoL was added for RNA isolation.

#### Endothelial cells.

Hearts were prepared as described for fibroblasts, however before plating, precoated CD31 antibody magnetic beads (Miltenyi Biotec, 130-097-418) were introduced to the suspension and incubated for 20 minutes before being immobilized by a magnet and the other cells washed away. Remaining cells were released from the beads, cultured on treated plates with EBM-2 cell media (w/ 10% FBS, 1% pen/strep, 0.1% ITS) for 24 h, followed by wash with PBS to remove dead cells and residual beads, followed by addition of triZoL for RNA isolation.

#### RNA isolation.

All RNA isolations were performed using the Zymogenetics Direct-zol RNA miniprep kit (R2052) according to manufacturer instructions. RNA quantity was measured using Qubit RNA High Sensitivity Assay (ThermoFisher, Q32855) and integrity determined using an Agilent Bioanalyzer. Only samples with RIN > 7.0 were used for library preparation, detailed below.

### Cardiac cell isolation for snRNAseq

On two occasions, hearts were excised from 3 littermates collected at P21. If 2 females and 1 male were used for the first collection, then the reverse was done on the second collection, such that the final sequencing represents 6 hearts, 3 males and 3 females.

Hearts were extracted from euthanized mice by cutting the aorta just below the arch arteries, along with the other major vessels. Isolated hearts were washed in ice cold Kruftbruhe (KB) solution and secured by their aortas to 18 or 20-gauge cannulas then tied off with a 3–0 silk suture. Atria were removed with Vannas micro spring scissors. Cannulated ventricles were then hung from a Langendorff apparatus and perfused with calcium-free Tyrode’s buffer, followed by 25–50 mL of 1 mg/mL collagenase type II (Thermo Fisher, 17101015) dissolved in calcium-free Tyrode’s buffer. Both solutions were warmed to 37°C. Following perfusion, ventricular tissue was diced with dissection scissors, triturated in ice cold Kruftbrühe (KB) solution using a wide bore 1 mL pipette, and allowed to settle for 10 minutes on ice.

The supernatant was removed, and the loose pellet was resuspended in 5 mL of Lysis buffer prepared as previously described [82], with only one adjustment – 50 µl of 10% Triton-X-100 was added (final concentration 0.1%). Cells were incubated in Lysis buffer + Triton for 5 minutes on ice, after which they were homogenized with a Tissue Tearor electric tissue homogenizer (Model # 985370) at the second lowest setting for 20–30 seconds and left to sit again for another 5 minutes on ice. They were then transferred through a 15 mL glass Dounce homogenizer and further homogenized with 20 strokes of the A pestle and 20 strokes of the B pestle. Homogenized cell suspensions were sequentially filtered through a 70 µM, 40 µm, and 20 µm cell strainer to remove debris and undigested materials. Samples were then spun at 1000G for 5 minutes and resuspended in 1 mL of 2% BSA dissolved in D-PBS with RNaseOut (Invitrogen, 200U/mL). A small aliquot was set aside to serve as an unstained control for fluorescent activated cell sorting (FACS). The remainder of the suspension was stained with DAPI at 10 µg/ml for 5 minutes on ice. Samples were spun at 1000G for 5 minutes and resuspended in fresh 2% BSA-RNaseOut solution.

Following staining, nuclei were sorted on a BD FACSMelody at 4°C. Following standard protocols, forward and side scatters were used to remove doublets. Unstained controls were used to set the V450 gate. 432,000 nuclei were collected into a 2 mL centrifuge tube preloaded with 500 µL of 2% BSA-RNaseOut solution. Sorted nuclei were spun down at 1000G for 5 minutes, supernatant was removed, and samples were resuspended in 100 µL of 2% BSA-RNaseOut solution (Invitrogen) before proceeding to 10x library preparation.

### Bulk and single nucleus RNAseq library preparation

*snRNAseq*: Nuclei were quantified with a Luna Fl cell counter (Logos Biosystems) and the volume was adjusted to obtain the ideal concentration of nuclei recommended by 10x Genomics (1000 nuclei/µL). Individual nuclei were paired with Chromium v3.1 gel beads and cDNA synthesis, barcoding, and dual index library preparation was performed using Chromium Next GEM V3.1 chemistry according to the manufacturer’s recommendation (10x Genomics). 10,000 nuclei were targeted for each sample with 13 cycles for cDNA amplification and 13 cycles for sample index PCR. The fragment size of cDNA and libraries was assessed using Agilent’s 5200 Fragment Analyzer System to verify product quality prior to sequencing with RIN > 7.0 used as a cutoff for sequencing.

*Bulk RNAseq of purified fractions*: Sequencing libraries were prepared using 3 μg total RNA from isolated RNA using the Stranded mRNA-Seq Kit (KAPA Biosystems).

*Bulk RNAseq of whole tissue*: mRNA-Seq libraries were constructed using 4 μg total RNA with the Stranded mRNA-Seq Kit (KAPA Biosystems) at Novogene (Sacramento, CA).

### RNA sequencing

*snRNAseq*: 2 libraries (3 mice per library) were sequenced at the Roy J. Carver Biotechnology Center at the University of Illinois, Urbana Champaign on a NovaSeq 6000 using one S4 lane with 2X150nt reads. Samples were demultiplexed and mapped to the mm10 genome using Cell Ranger v6.1.1 (10X Genomics).

*Bulk RNAseq of purified fractions*: Bulk RNA fractions were sequenced 2x50 (paired end) on a Novaseq 6000 S2 chip at the Technology Center for Genomics and Bioinformatics (TCGB) at UCLA.

*Bulk RNAseq of whole tissue*: Samples were multiplexed with Illumina TruSeq adapters and run on a single 75-cycle paired end sequencing run with an Illumina NextSeq-500.

All bulk RNAseq samples were demultiplexed, then decoy-aware pseudo-aligned to the GRCm39 transcriptome with Salmon v1.10.2 [83] and read quality was checked with FastQC v0.12.1.[84] and summarized with MultiQC [85]. Then, samples were joined into a counts matrix with the txmeta [86] and tximport [87] packages in R version 4.3.1. Bulk RNAseq and snRNAseq data are publicly available as NCBI BioProjects under accessions PRJNA1122769 (bulk RNAseq) and PRJNA880279 (snRNAseq). The full Snakemake [88] analysis pipeline is publicly available on GitHub (https://github.com/guralbrian/bulk_decon).

### Single nucleus RNAseq data analysis

Raw counts, barcode, and feature matrices were joined into a single Seurat object [89] for each replicate. Ambient droplet RNA and doublets were identified and removed *in-silico* with DropletUtils::emptyDrops [90] and scDblFinder::computeDoubletDensity [91], respectively. Sample replicates were merged into a single Seurat object, then filtered by feature count, transcript count, mitochondrial transcript percentage, and doublet score. Raw counts were normalized, scaled, applied to principal component analysis, and integrated by harmony::RunHarmony [92]. For clustering, the number of principal components included was determined by finding the first PC which exhibits cumulative percent variability greater than 90% or that which explains less than 5% of the total variability. Samples were then clustered by the standard Seurat methods, using the *harmony* reduction. See S2 Table for detailed transcriptional marker list.

We converted Ensembl IDs to gene symbols to match the bulk and snRNA-seq formats; among duplicate gene symbols, we kept the one with the highest average gene expression.

Before performing deconvolution, the 35,334 unique transcripts from the bulk RNAseq data and 19,883 from the snRNAseq data were subset to the 15,376 genes present in both datasets. Then, scran::findMarkers [25] was used to find markers for each Seurat cluster. Markers were ordered by adjusted p-value and the top 15 markers per cluster were retained. After, marker genes were manually queried in ToppGene [93] for associations with known cell types. High confidence cell type associations were annotated to the relevant cluster and small clusters or those with low confidence annotated were excluded from further analysis. After, five cell-type clusters were retained: endothelial cells, cardiomyocytes, fibroblasts, a joint vascular smooth muscle cell and pericyte cluster, a joint monocyte and macrophage cluster, and smooth muscle cells.

### Deconvolution analysis

Deconvolution of all bulk RNAseq datasets was performed with MuSiC [94], an R package for deconvolution of bulk RNA sequencing data which uses single cell or single nucleus RNA sequencing data as a reference. Only genes present in the 15 markers per cell type identified with scran (S2 Table) were included in deconvolution analysis. Raw expression counts were applied to MuSiC::music_prop and proportion estimates from the weighted deconvolution were used in all further analysis.

### Dirichlet model

To model the relationship between treatment or genotype with estimated cell type proportions, we used a Dirichlet regression model. The DirichReg function from the DirichletReg [34] package was run with a genotype-treatment interaction term with common parameterization.

### Differential expression

Differential expression analysis was performed with DESeq2(19). Two iterations were performed: without covariates, and with covariates including cardiomyocyte and fibroblast proportions. The DESeq2 workflow included creating DESeqDataSet objects, filtering genes with more than 10 reads in at least four samples. Both iterations modeled gene expression as the product of the additive effects of genotype, treatment, and their interaction, with the second analysis iteration also including additive effects of each cell type proportion. Multiple testing correction was performed using a false discovery rate (FDR) threshold of 0.05 and effects of low-coverage transcripts were minimized by the lfcShrink function [95]. See S1 Table for full DESeq2 outputs.

DESeq2 results were validated with limma-voom [96,97] Raw counts were filtered to retain genes with at least 1 transcript per million (TPM) in at least 4 samples, normalized using calcNormFactors, and transformed with voom to account for mean-variance relationships. We created two linear models: one with genotype × treatment interaction terms alone, and another that additionally included CLR-transformed cell type proportions as covariates. Empirical Bayes moderation was applied using eBayes, and the same contrasts examined in the DESeq2 analysis were tested.

DESeq2 and limma-voom exhibit high concordance when DESeq2 is not run with lfcShrink, with logFC of all transcripts in the genotype-by-treatment contrast exhibiting a Spearman correlation coefficient of 0.77 before cell types were included in the model and 0.971 after cell types were added to the model (S4 Fig). Shrinkage reduces spurious correlations of low-abundance genes by accordingly adjusting their estimated log-fold changes [95] and mildly reduces the correlation coefficients between the two approaches to 0.701 and 0.892 for pre- and post-cell type inclusion.

### Simulated differential expression

Bulk RNAseq samples were simulated with predefined cell type contributions by leveraging our snRNAseq dataset. First, for each cell type cluster (cardiomyocytes, fibroblasts, endothelial cells, macrophages, and pericytes/smooth muscle cells), we generated a transcriptomic profile by summing the expression counts for all nuclei in the cluster and normalizing by the total counts. This produced a probability distribution for each gene representing the likelihood of being selected from that cell type.To mimic typical bulk RNAseq data, we sampled transcripts from these distributions to yield a total of 25 million reads per sample [98] (i.e., a sample with 50% cardiomyocytes would sample the expression profile of the cardiomyocyte cluster 12.5 million times). To systematically investigate the impact of cellular composition on differential expression, we simulated 81 distinct conditions in which the proportion of cardiomyocytes varied from 30% to 70% in stepwise changes of 0.5% CM proportion to yield 81 groups ((70–30)/0.5 + 1 = 81). For each replicate sample within these conditions, proportions for fibroblasts and macrophages were adjusted in anti-correlation with the cardiomyocyte change (increasing as cardiomyocytes decreased, based on estimated factors derived from experimental observations), while the remaining proportion was distributed amongst other minor cell types based on reference baseline estimates. Random noise was added to these target proportions before normalizing the final composition vector for each sample to sum to one. Additionally, we doubled the expression of a set of 10% genes to serve as composition-independent expression positive controls (S2 Fig). For samples not belonging to the baseline (50% cardiomyocyte) group, the expression of these designated true positive genes was altered by applying fold changes drawn from a log-normal distribution (mean log2FC ≈ 0.58 [equivalent to 1.5x linear fold change], SD log2FC = 0.5). To test the effect of including composition, DESeq2 was run with two models:


gene expression ~ 0 + sample group

gene expression ~ 0 + sample group + major cell type abundance 

For each model, sample groups were contrasted against the 50% cardiomyocytes group and the number of significantly differentially expressed genes was recorded. The analysis was repeated with a total of three different representations of cell type proportions in the model: untransformed proportions, centered-log-ratios of proportions (a transformation common in compositional statistics which divides each compositional part by the geometric mean of all parts to reduce co-correlation), and principal components from principal component analysis of cell type proportions. All simulated differential expression analysis were conducted in batches of 1000 genes. Model performance in accurately identifying the simulated differentially expressed genes was evaluated using the F1 score, calculated as the harmonic mean of precision (the fraction of predicted positive genes that were true positives) and recall (the fraction of true positive genes that were correctly identified), with higher scores indicating better classification accuracy.

### GSEA analysis

Expression changes of individual genes were summarized into biological pathways by gene ontology (GO) enrichment using clusterProfiler v4.10.0 [23]. Gene names were converted to ENSEMBL IDs using the org.Mm.e.g.,db reference package v3.18.0 [99]. For each model and variable, all genes were ordered by their –log10(adjusted P-value) multiplied by the sign (i.e., positive or negative) of the log-fold change. These were provided to clusterProfiler::gseGO using biological pathway ontology and Benjamini-Hochberg p-value adjustment. See S4 Table for entire GSEA term dataset.

### Figure generation

Schematics and diagrams (Figs 1A, 2A, 3A, 3B, 4A and 4B) were produced with Biorender. All other plots were generated using the following R packages: dot and bar plots were produced with ggplot2 [100], upset plots with ComplexUpset [101,102], UMAP plot with ggplot2 and tidyseurat [103], ternary plot with ggtern [104], and tables with gt [105] and gtExtras [106].

## Supporting information

S1 Fig UMAP of single nucleus RNA sequencing dataset before cluster annotation and removal. snRNAseq quality control involved doublet detection, ambient RNA removal, filtering by features such as gene counts and mitochondrial transcript abundance. Clusters 6–10 were excluded from the final analysis due to their low nuclei counts and poor annotation to known cell types. Clusters 3 and 5 were merged due to their similar marker profiles.
(PDF)

S2 Fig Comparison of methods for representing cellular proportions in DESeq2. Mean F1 score (points) ± standard deviation (error bars) for detecting simulated differentially expressed genes using DESeq2. Different models incorporating cell type abundance were tested: no cell type adjustment (‘No cell types’), raw cell type proportions (‘Raw prop’), centered log-ratio (CLR) transformed proportions (‘CLR’), and the first principal component of proportions (‘PC1’). Models labeled ‘1x’ included only cardiomyocyte abundance; models labeled ‘2x’ included both cardiomyocyte and fibroblast abundance as covariates. Performance is evaluated separately for conditions simulated with major compositional changes (>10% shift relative to the 50% cardiomyocyte baseline group, right panel) and minor changes (<10% shift, left panel). The F1 score measures the accuracy of differential expression calls (balancing precision and recall), with higher values indicating better performance. Note the substantial improvement of all correction methods (Raw prop, CLR, PC1) over the ‘No cell types’ model. In this simulation, the PC1 model achieved the highest F1 score. Data were generated from simulated bulk RNA-seq experiments with known ground truth differential expression status across varying cardiomyocyte proportions and correlated cell type shifts.
(PDF)

S3 Fig Area occupied by sarcomeric actinin in immunohistology staining of cardiac cross sections. Five representative regions were evaluated in each zone from Figure 5B, and modest variation is seen within each sample between regions. The area was measured in pixel counts.
(PDF)

S4 Fig Reproducibility of results by limma-voom and DESeq2. (PDF)

S1 Table DESeq2 differential expression analysis results. Full output of DESeq2 analysis showing results from both uncorrected and cell-type-corrected models, including log2 fold changes, p-values, and adjusted p-values for all genes across different comparisons.
(CSV)

S2 Table Cell type-specific marker genes used for deconvolution. List of 15 marker genes per cell type identified by scran and used as input for MuSiC deconvolution analysis.
(CSV)

S3 Table Summary of differential expression changes after cell-type abundance correction. Table summarizing the number of differentially expressed genes (DEGs) before (Uncorrected) and after (Corrected) adjusting for cell abundance, and the number of genes gaining or losing significance by more than one order of magnitude (OM) or crossing the significance threshold (Padj = 0.05).
(CSV)

S4 Table Gene set enrichment analysis results. Full GSEA results for all genes across all variables and DESeq2 model iterations.
(CSV)

S5 Table RNAscope probes and reagents. List of probes and control probes used for RNAscope in situ hybridization experiments.
(XLSX)

S6 Table Parameters of RNAscope Image Quantification. All relevant settings needed to reproduce the image quantifications. Includes results of our own quantification as well as the settings used to produce them.
(XLSX)
