# Supplementary figures and images for: Novel insights into post-myocardial infarction cardiac remodeling through algorithmic detection of cell-type composition shifts
Source: PLoS Genet. 2025 Jul 24;21(7):e1011807. doi: 10.1371/journal.pgen.1011807 (PMC12309993; doi:10.1371/journal.pgen.1011807)

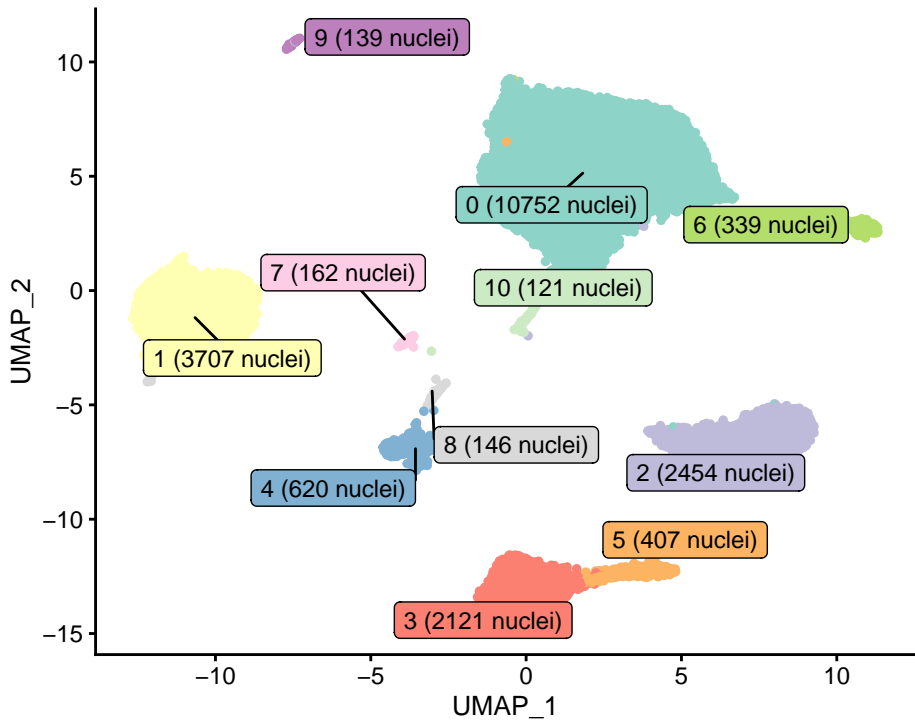

Supplement: S1 Fig — Clusters 6–10 were excluded from the final analysis due to their low nuclei counts and poor annotation to known cell types. Clusters 3 and 5 were merged due to their similar marker profiles. (PDF) [file pgen.1011807.s001.pdf]

F1 Score

Major change ( $>10\%$  shift)

Minor change ( $<10\%$  shift)

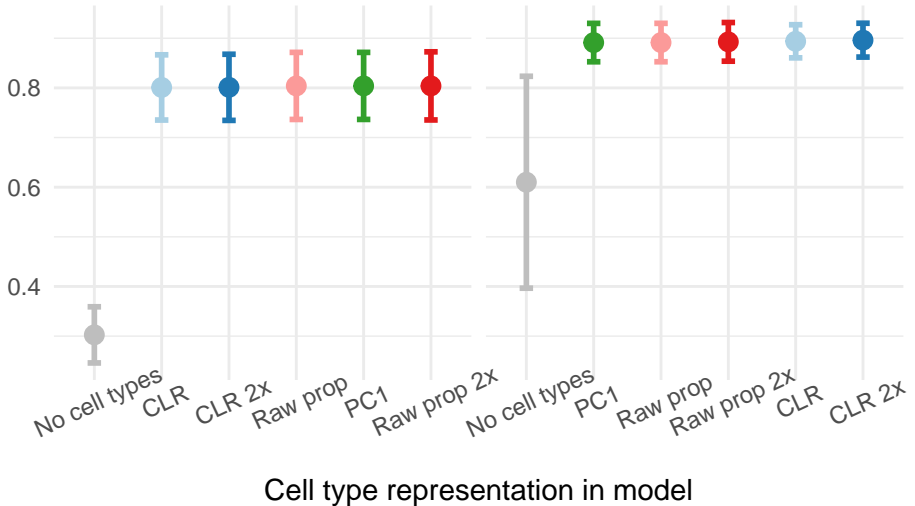

Supplement: S2 Fig — Mean F1 score (points) ± standard deviation (error bars) for detecting simulated differentially expressed genes using DESeq2. Different models incorporating cell type abundance were tested: no cell type adjustment (‘No cell types’), raw cell type proportions (‘Raw prop’), centered log-ratio (CLR) transformed proportions (‘CLR’), and the first principal component of proportions (‘PC1’). Models labeled ‘1x’ included only cardiomyocyte abundance; models labeled ‘2x’ included both cardiomyocyte and fibroblast abundance as covariates. Performance is evaluated separately for conditions simulated with major compositional changes (>10% shift relative to the 50% cardiomyocyte baseline group, right panel) and minor changes (<10% shift, left panel). The F1 score measures the accuracy of differential expression calls (balancing precision and recall), with higher values indicating better performance. Note the substantial improvement of all correction methods (Raw prop, CLR, PC1) over the ‘No cell types’ model. In this simulation, the PC1 model achieved the highest F1 score. Data were generated from simulated bulk RNA-seq experiments with known ground truth differential expression status across varying cardiomyocyte proportions and correlated cell type shifts. (PDF) [file pgen.1011807.s002.pdf]

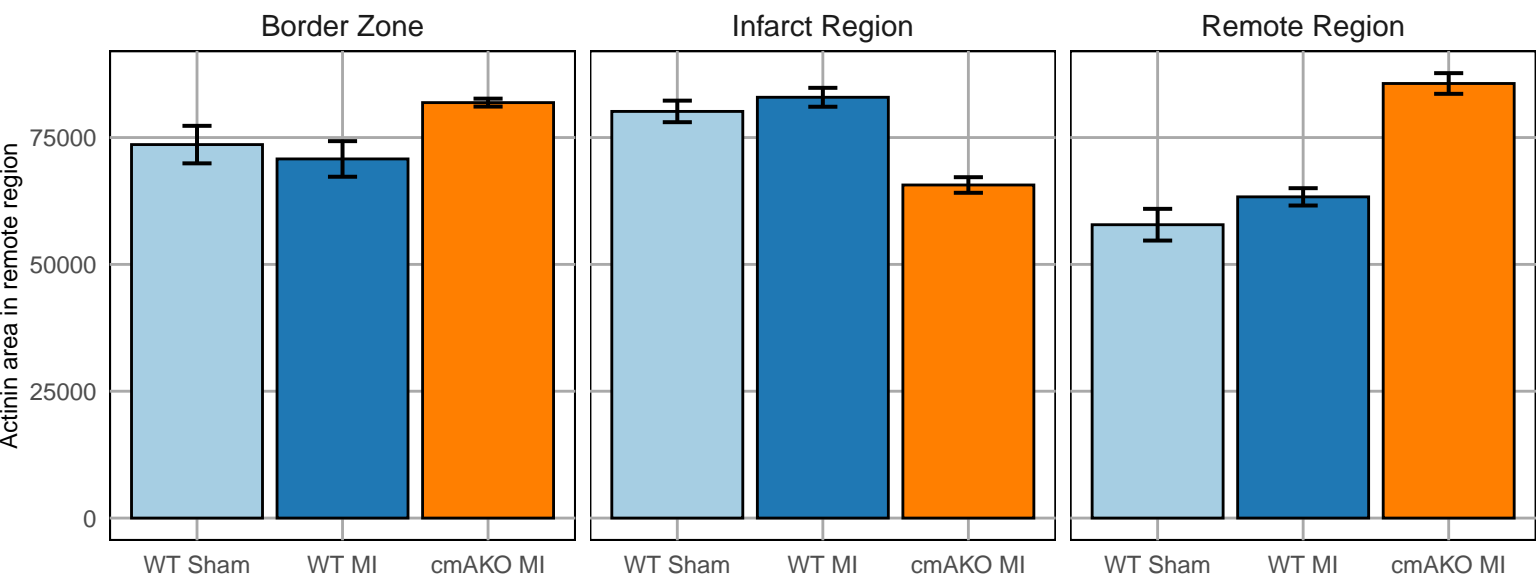

Supplement: S3 Fig — Five representative regions were evaluated in each zone from Figure 5B, and modest variation is seen within each sample between regions. The area was measured in pixel counts. (PDF) [file pgen.1011807.s003.pdf]

## IfcShrink applied to DESeq

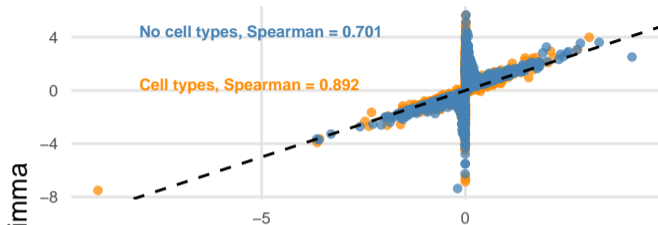

## No shrinkage applied

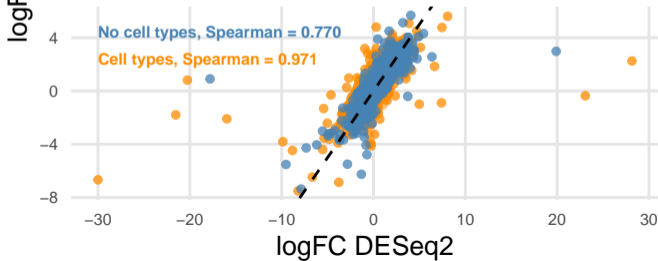

Supplement: S4 Fig — (PDF) [file pgen.1011807.s004.pdf]
